# A stepwise strategy integrating dynamic stress CT myocardial perfusion and deep learning–based FFR_CT_ in the work-up of stable coronary artery disease

**DOI:** 10.1007/s00330-023-10562-x

**Published:** 2024-01-12

**Authors:** Lijuan Lyu, Jichen Pan, Dumin Li, Dexin Yu, Xinhao Li, Wei Yang, Mei Dong, Yeming Han, Yongfeng Liang, Pengfei Zhang, Mei Zhang

**Affiliations:** 1 The Key Laboratory of Cardiovascular Remodeling and Function Research, Chinese Ministry of Education, Chinese National Health Commission and Chinese Academy of Medical Sciences, The State and Shandong Province Joint Key Laboratory of Translational Cardiovascular Medicine, Department of Cardiology, Qilu Hospital, Cheeloo College of Medicine, Shandong University, Jinan, Shandong People’s Republic of China; 2 Department of Radiology, Qilu Hospital, Cheeloo College of Medicine, Shandong University, Jinan, Shandong People’s Republic of China

**Keywords:** Coronary artery disease, Computed tomography myocardial perfusion imaging, Myocardial blood flow, Computed tomography–derived flow fractional reserve, Stepwise strategy

## Abstract

**Objectives:**

To validate a novel stepwise strategy in which computed tomography–derived fractional flow reserve (FFR_CT_) is restricted to intermediate stenosis on coronary computed tomography angiography (CCTA) and computed tomography myocardial perfusion imaging (CT-MPI) was reserved for vessels with gray zone FFR_CT_ values.

**Materials and methods:**

This retrospective study included 87 consecutive patients (age, 58 ± 10 years; 70% male) who underwent CCTA, dynamic CT-MPI, interventional coronary angiography (ICA), and fractional flow reserve (FFR) for suspected or known coronary artery disease. FFR_CT_ was computed using a deep learning–based platform. Three stepwise strategies (CCTA + FFR_CT_ + CT-MPI, CCTA + FFR_CT_, CCTA + CT-MPI) were constructed and their diagnostic performance was evaluated using ICA/FFR as the reference standard. The proportions of vessels requiring further ICA/FFR measurement based on different strategies were noted. Furthermore, the net reclassification index (NRI) was calculated to ascertain the superior model.

**Results:**

The CCTA + FFR_CT_ + CT-MPI strategy yielded the lowest proportion of vessels requiring additional ICA/FFR measurement when compared to the CCTA + FFR_CT_ and CCTA + CT-MPI strategies (12%, 22%, and 24%). The CCTA + FFR_CT_ + CT-MPI strategy exhibited the highest accuracy for ruling-out (91%, 84%, and 85%) and ruling-in (90%, 85%, and 85%) functionally significant lesions. All strategies exhibited comparable sensitivity for ruling-out functionally significant lesions and specificity for ruling-in functionally significant lesions (*p* > 0.05). The NRI indicated that the CCTA + FFR_CT_ + CT-MPI strategy outperformed the CCTA + FFR_CT_ strategy (NRI = 0.238, *p* < 0.001) and the CCTA + CT-MPI strategy (NRI = 0.233%, *p* < 0.001).

**Conclusions:**

The CCTA + FFR_CT_ + CT-MPI stepwise strategy was superior to the CCTA + FFR_CT_ strategy and CCTA+ CT-MPI strategy by minimizing unnecessary invasive diagnostic catheterization without compromising the agreement rate with ICA/FFR.

**Clinical relevance statement:**

Our novel stepwise strategy facilitates greater confidence and accuracy when clinicians need to decide on interventional coronary angiography referral or deferral, reducing the burden of invasive investigations on patients.

**Key Points:**

*• A stepwise CCTA + FFR*_*CT*_ *+ CT-MPI strategy holds promise as a viable method to reduce the need for invasive diagnostic catheterization, while maintaining a high level of agreement with ICA/FFR.*

*• The CCTA + FFR*_*CT*_ *+ CT-MPI strategy performed better than the CCTA + FFR*_*CT*_ *and CCTA + CT-MPI strategies.*

*• A stepwise CCTA + FFR*_*CT*_ *+ CT-MPI strategy allows to minimize unnecessary invasive diagnostic catheterization and helps clinicians to referral or deferral for ICA/FFR with more confidence.*

**Supplementary Information:**

The online version contains supplementary material available at 10.1007/s00330-023-10562-x.

## Introduction

Accurate diagnostic tests are prerequisite for identifying patients suitable for revascularization. Fractional flow reserve (FFR) is the gold standard for guiding revascularization [1, 2]. However, the use of FFR remains low due to its invasive nature and prohibitive costs [3, 4]. Guidelines recommend noninvasive testing for patients with suspected myocardial ischemia before invasive procedures [5–7].

Coronary computed tomography angiography (CCTA) has become a robust tool in ruling-out coronary artery disease (CAD) given its high negative predictive value (NPV) [8–11]. Recently, updated guidelines recommend CCTA as the first-line test for patients with suspected CAD [5–7]. However, CCTA cannot assess the physiological significance of coronary artery stenosis. Hence, computed tomography–derived fractional flow reserve (FFR_CT_) and computed tomography myocardial perfusion imaging (CT-MPI) have been introduced as novel functional imaging tools to overcome the inherent CCTA drawbacks.

Benefiting from the fact that it can compute from standard CCTA images without requiring additional image acquisition or vasodilator application, FFR_CT_ holds the potential to be the first choice for hemodynamic assessment after CCTA. Moreover, on-site FFR_CT_ (machine learning– or deep learning–based algorithms) computation requires only several minutes, and has shown good agreement with invasive FFR and HeartFlow FFR_CT_ [12, 13]. Multicenter clinical trials have confirmed that FFR_CT_ can improve the accuracy of diagnosis for vessel-specific ischemia [14–17], and reduce the need for further noninvasive and invasive testing [17, 18]. However, several unresolved issues for clinical implementation of FFR_CT_ persist. Several studies confirmed that the accuracy of FFR_CT_ is disturbingly low (46 to 68%) within a gray zone (approximately 0.80), and the proportion of patients exhibiting such gray zone FFR_CT_ values is non-trivial [19–22]. Despite those facts, few studies have addressed how to deal with such ambiguous situation when the FFR_CT_ value fell within the gray zone in clinical practice. Additionally, meta-analyses and reviews based on HeartFlow FFR_CT_ and reduced-order computational fluid dynamic–based FFR_CT_ have reported FFR_CT_ gray zone ranges of 0.74–0.82 and 0.75–0.84 [21, 23]. Currently, there is no report on the gray zone of deep learning–based FFR_CT_.

CT-MPI showed comparable diagnostic accuracy to that of FFR_CT_ in detecting vessel-specific ischemia [22, 24]. CCTA + CT-MPI–guided patient management is a promising approach for reducing unnecessary invasive procedures [25]. However, CT-MPI is time-consuming and requires additional radiation exposure and contrast agent. The place of CT-MPI in the diagnostic workflow of CAD remains to be discussed.

Based on the above evidence, we here proposed a CCTA + FFR_CT_ + CT-MPI stepwise strategy, in which FFR_CT_ was performed only in intermediate coronary stenosis on CCTA, and CT-MPI was subsequently used to identify vessel-specific ischemia when FFR_CT_ values fell within the gray zone. This stepwise strategy is potentially to provide a solution for FFR_CT_ “gray zone.” Therefore, we hypothesized that the CCTA + FFR_CT_ + CT-MPI stepwise strategy would be superior to the CCTA + FFR_CT_ and CCTA + CT-MPI strategies by minimizing unnecessary invasive procedures.

## Materials and methods

### Study population

**Fig. 1:**
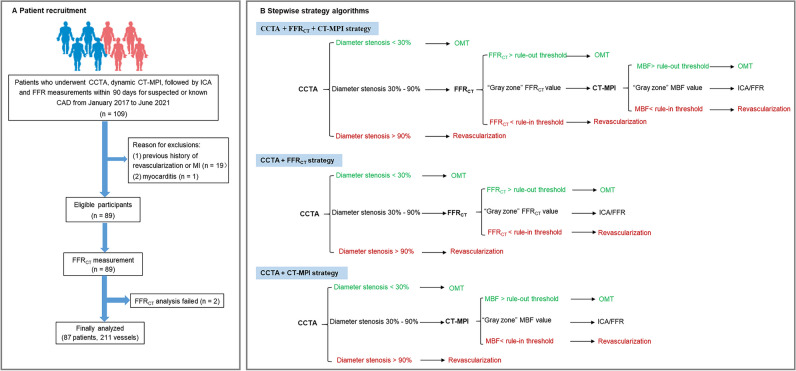
**A, B** Flowchart of patient recruitment and stepwise strategy algorithms. CAD, coronary artery disease; CCTA, coronary computed tomography angiography; CT-MPI, computed tomography myocardial perfusion imaging; FFR_CT_, computed tomography–derived flow fractional reserve; FFR, fractional flow reserve; ICA, invasive coronary angiography; MI, myocardial infarction; OMT, optimal medical treatment

This study complies with the Declaration of Helsinki. The study protocol was approved by the local hospital ethical committees. We retrospectively enrolled 87 consecutive patients with suspected or known CAD from a single center between January 2017 and June 2021. The inclusion criteria were as follows: (1) patients with suspected or known CAD who underwent CCTA, stress dynamic CT-MPI, and FFR_CT_ ≤ 90 days before interventional coronary angiography (ICA); (2) clinically indicated ICA was performed according to current clinical standards and guidelines, regardless of CT-MPI and FFR_CT_ findings; (3) availability of complete imaging and clinical data. The exclusion criteria included the following: (1) low pre-test likelihood of CAD (< 15%); (2) previous history of coronary revascularization or myocardial infarction; (3) acute coronary syndrome or clinical instability; (4) nonischemic cardiomyopathy; (5) atrial fibrillation; (6) nondiagnostic CT image quality. Fig. 1A shows the flow diagram of the study.

### CCTA and CT-MPI protocol

All participants were tested with a comprehensive protocol integrating CCTA and dynamic CT-MPI by using a third-generation dual-source computed tomography scanner (SOMATOM Force; Siemens). The detailed protocol and scan parameters are provided in the Supplemental Methods. In brief, dynamic CT-MPI was started after a 3-min continuous adenosine infusion at a rate of 140 µg/kg/min. Dynamic CT-MPI images were acquired in the end-systolic phase in shuttle mode. Nitroglycerin was given sublingually to all participants 5 min after CT-MPI. Subsequently, a bolus of contrast media was injected into the antecubital vein at a rate of 4–5 mL/s. CCTA image was acquired using the retrospective electrocardiography-triggered acquisition mode. Using a constant conversion coefficient of 0.026 [26], the effective radiation dose was 13.79 (11.31–16.29) mSv for CCTA and 7.74 (5.70–9.30) mSv for dynamic CT-MPI.

### CCTA and CT-MPI analysis

The details of CCTA and CT-MPI image analysis are provided in the Supplemental Methods. All the CCTA and CT-MPI images were evaluated by independent readers who were blinded to other examination results and clinical information. The CCTA images were independently analyzed on an offline workstation (Syngo Via, Siemens) by two experienced radiologists. Segmental analysis of the coronary arteries was performed for arteries > 1.5 mm in diameter. The degree of stenosis was reported as the percentage decrease in lumen diameter.

The CT-MPI images were processed with the CT-MPI software package (VPCT, Siemens). Quantification of myocardial blood flow (MBF) was performed using a hybrid deconvolution model, as previously reported [22]. To calculate the MBF, a region of interest (ROI) was manually placed on a short-axis view on a per-segment basis according to the American Heart Association (AHA) 17-segment model [27].

### Machine learning*–*based FFR_CT _assessments

FFR_CT_ were calculated using commercially available software (DEEPVESSEL FFR, Keya Medical), which was based on machine learning (ML) methods that trained using a deep learning framework. Details of the FFR_CT_ algorithm are provided in the Supplemental Methods. Computation of FFR_CT_ was performed in a blinded manner by the core laboratory of Keya Medical. FFR_CT_ results were returned to researchers for blinded analysis. Clinicians were blinded to FFR_CT_ results so as not to interfere with decision-making. The lesion-specific FFR_CT_ values were measured 20 mm distal to the stenosis. For multiple lesions of the same vessel, the lesion with the lowest FFR_CT_ value was recorded.

### ICA and invasive FFR assessments

ICA was performed with standard methods. All coronary arteries and main branches were evaluated by two interventional cardiologists. Lesions with luminal stenosis between 30 and 90% were referred for invasive FFR measurements. During steady-state hyperemia, FFR was measured using a 0.014-inch pressure guidewire (Prime Wire Prestige PLUS, Volcano Corporation). Hyperemia was induced by an intravenous infusion of adenosine at 140 mg/kg/min. Functionally ischemic lesions were defined as lesions with more than 90% stenosis or an FFR ≤ 0.80. Nonischemic lesions were defined as lesions with less than 30% stenosis or an FFR > 0.80.

### “Gray zone”for FFR_CT _and MBF

Gray zone thresholds for FFR_CT_ and MBF were calculated through receiver-operating characteristic (ROC) analysis. The rule-out threshold was derived from a predefined NPV > 95% and rule-in threshold from a predefined positive predictive value (PPV) > 95% for the gold standard diagnosis of functional ischemia. The “gray zone” was defined as values between the rule-in and rule-out thresholds.

### CCTA + FFR_CT _+ CT-MPI stepwise strategy

As shown in Fig. 1B, if all coronary stenoses were < 30% on CCTA, optimal medical treatment (OMT) was indicated; while if at least one coronary stenosis was > 90%, ICA and revascularization were indicated. FFR_CT_ was indicated if at least one coronary stenosis was between 30 and 90% on CCTA. Further triage will follow FFR_CT_: (a) FFR_CT_ > the rule-out threshold, OMT was indicated; (b) FFR_CT_ < the rule-in threshold, revascularization was indicated; (c) FFR_CT_ fell within the “gray zone,” CT-MPI was indicated before invasive assessment. Triage was based on CT-MPI: (a) MBF > the rule-out threshold, OMT was indicated; (b) MBF < the rule-in threshold, revascularization was indicated; (c) MBF fell within the “gray zone,” invasive assessment was indicated before revascularization.

### CCTA + FFR_CT _and CCTA + CT-MPI stepwise strategy

CCTA + FFR_CT_ and CCTA + CT-MPI strategies were similar to the diagnostic algorithm proposed by Hecht et al [26]. As depicted in Fig. 1B, OMT was prescribed in cases with all coronary stenoses < 30% and revascularization was prescribed in cases with at least one coronary stenosis > 90%. When at least one coronary stenosis was between 30 and 90%, FFR_CT_ (or CT-MPI) was prescribed. OMT was indicated for cases with FFR_CT_ (or MBF) > the rule-out threshold, and revascularization was indicated for cases with FFR_CT_ (or MBF) < the rule-out threshold. Invasive FFR was indicated before revascularization if FFR_CT_ (or MBF) fell within the “gray zone.”

### Statistical analysis

Normality of the data distribution was tested by the Kolmogorov–Smirnov test. Normally distributed continuous variables are described as mean ± standard deviation and were compared using Student’s *t*-test. Non-normally distributed continuous variables are described as medians (interquartile range [IQR]) and compared using the Mann–Whitney *U* test. Categorical variables were described as number (proportion) and were compared using the *χ*^2^ test or Fisher’s exact test. Correlation between FFR_CT_ and invasive FFR was evaluated using Spearman’s correlation coefficients, and the agreement between FFR_CT_ and invasive FFR was assessed by a Bland–Altman plot. For the stepwise strategy, the sensitivity, specificity, PPV, and NPV were calculated using two approaches: (i) rule-out approach of considering both the gray zone and ischemia categories as “positive,” and (ii) rule-in approach of considering only the ischemia category as “positive.” ROC curve analysis was performed for each stepwise strategy using ICA/FFR as the reference standard, and the area under ROC curve (AUC) and partial AUC were used to evaluate the performance of each strategy based on the rule-in and rule-out criteria. AUCs were compared using the Delong test. The net reclassification improvement (NRI) was calculated to ascertain the superior model [28, 29]. The 95% confidence intervals of the NRI were estimated by bootstrapping with 1000 iterations.

A two-sided *p* < 0.05 was considered statistically significant. Statistical analyses were performed using the MedCalc software package (MedCalc 15.2.0) and R (R statistics), version 4.0.4.

## Results

### Baseline characteristics

A total of 87 patients (age, 58 ± 10 years; 70% male) with 211 vessels were included in the study (Fig. 1A; Table 1). Seventy-nine vessels (37%) were identified as hemodynamically significant by ICA/FFR.

**Table 1:** Baseline characteristics of the study population

Parameter	
Number of patients, *n*	87
Number of vessels, *n*	211
LAD, *n* (%)	70 (33)
LCX, *n* (%)	70 (33)
RCA, *n* (%)	71 (34)
Age, years (mean ± SD)	58.1 ± 9.7
Male gender (%)	61 (70)
BMI, kg/m^2^ (mean ± SD)	26.0 ± 2.9
Coronary risk factors	
Hypertension, *n* (%)	60 (69)
Dyslipidemia, *n* (%)	78 (90)
Diabetes, *n* (%)	18 (21)
Smoking,* n* (%)	48 (55)
Family history of CAD, *n* (%)	16 (18)
Pre-test likelihood of CAD (%) ^†^	
15–65, *n* (%)	50 (57)
65–85, *n* (%)	34 (39)
> 85, *n* (%)	3 (3)
Diameter stenosis by CCTA (%)	
< 30, *n* (%)	76 (36)
30–90, *n* (%)	119 (56)
≥ 90, *n* (%)	16 (8)
Prevalence of obstructive CAD (≥ 50%) at ICA	
No disease, *n* (%)	16 (18)
1-vessel disease, *n* (%)	39 (45)
2-vessel disease, *n* (%)	17 (20)
3-vessel disease, *n* (%)	15 (17)
Patients, *n* (%)	71 (82)
Functionally Ischemic vessels*	79 (37)

*SD*, standard deviations; *BMI*, body mass index; *CAD*, coronary artery disease; *CCTA*, coronary computed tomography angiography; *ICA*, invasive coronary angiography; *LAD*, left anterior descending coronary artery; *LCX*, left circumflex coronary artery; *RCA*, right coronary artery. †Calculated by using the Diamond and Forrester Chest Pain Prediction Rule. *Diameter stenosis > 90% by ICA or invasive FFR < 0.8 in intermediate stenosis of 30 to 90%

### Characteristics of FFR_CT_ and dynamic CT-MPI

**Fig. 2:**
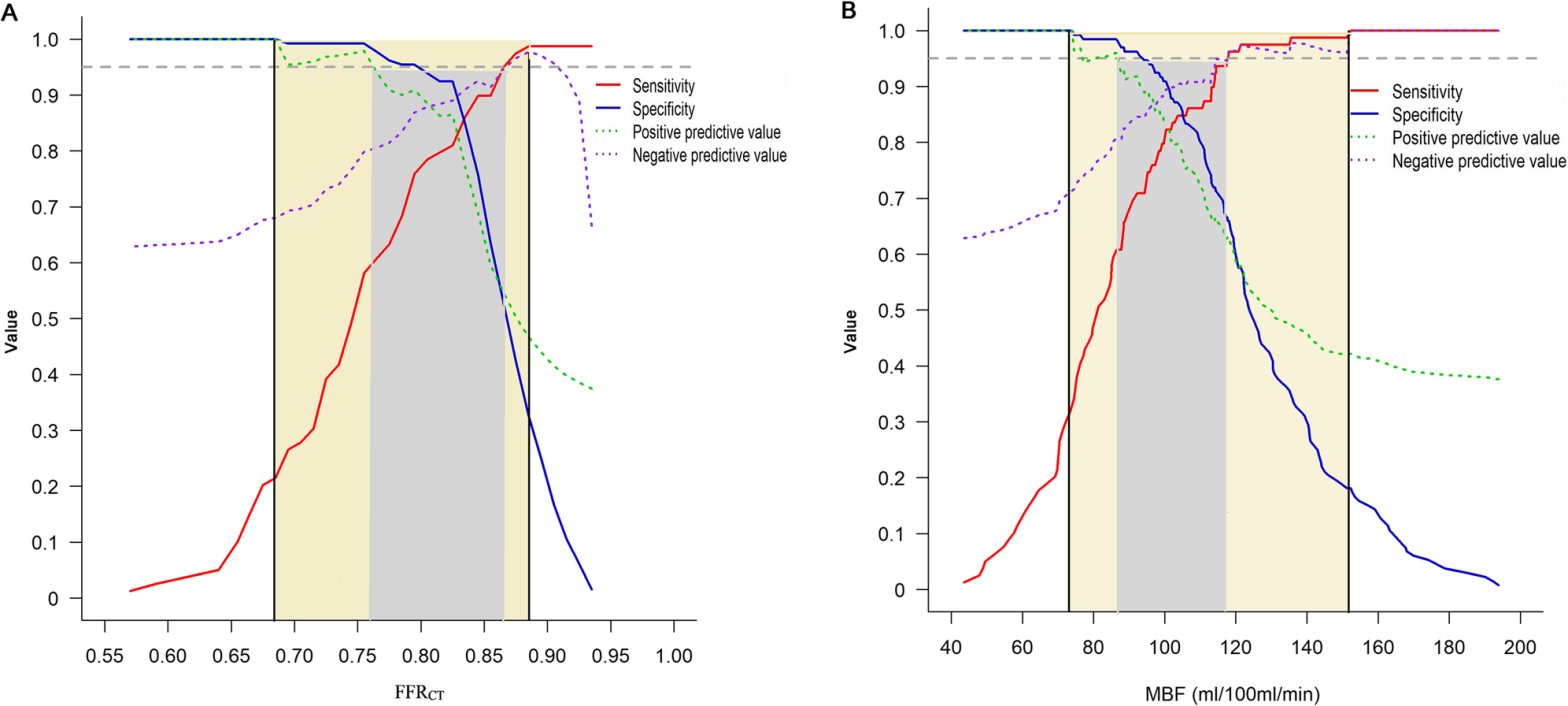
Sensitivity, specificity, NPV, and PPV of FFR_CT_ and MBF for prediction of functional ischemia. **A** To achieve a PPV and NPV of at least 95% at each extreme, the overall FFR_CT_ range was restricted to ≤ 0.75 and > 0.87 (gray area). FFR_CT_ values lower than 0.68 resulted in a PPV of 100%, and FFR_CT_ values higher than 0.88 resulted in an NPV of 98% (yellow area). **B** With the threshold for PPV and NPV set at 95%, the MBF range was restricted to < 86 mL/100 mL/min and > 118 mL/100 mL/min (gray area). To achieve a PPV and NPV of 100% at each extreme, the MBF values were restricted to ≤ 74 mL/100 mL/min and > 152 mL/100 mL/min (yellow area). FFR_CT_, computed tomography–derived flow fractional reserve; MBF, myocardial blood flow; NPV, negative predictive value; PPV, positive predictive value

In the per-vessel analysis, for each imaging modality, the highest frequency of false-positive and false-negative cases was clustered around the cutoff value (Figure S1). For vessels with invasive FFR measurements, FFR_CT_ was moderately correlated with FFR, with a Spearman correlation coefficient of 0.67 (95%CI 0.43 to 0.84; *p* < 0.001) (Supplementary Figure S2A). Bland–Altman plots showed slightly systematic overestimation of FFR by FFR_CT_ with a mean difference of 0.03 (limits of agreement, − 0.20 to 0.26) (Supplementary Figure S2B). To achieve a PPV and NPV of at least 95% at each extreme, the gray zone of FFR_CT_ and MBF values were 0.76–0.86 and 86–118 mL/100 mL/min, respectively (Fig. 2A, B).

### Diagnostic performance of the CCTA + FFR_CT_ + CT-MPI stepwise strategy

**Fig. 3:**
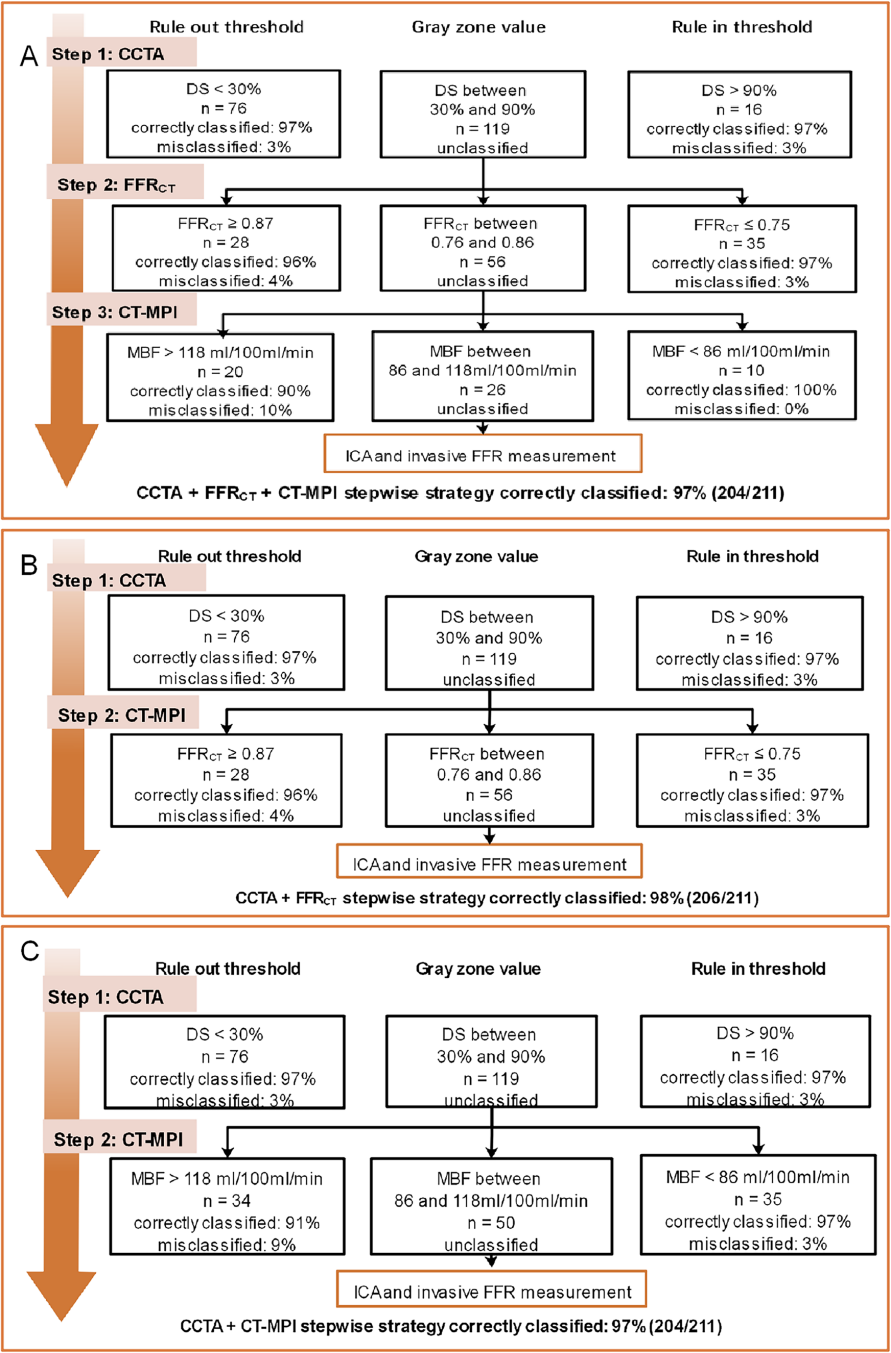
**A**–**C** Algorithm flowchart of the stepwise strategy. CCTA, coronary computed tomography angiography; CT-MPI, computed tomography myocardial perfusion imaging; DS, diameter stenosis; FFR_CT_, computed tomography–derived flow fractional reserve; FFR, fractional flow reserve; ICA, invasive coronary angiography

**Fig. 4:**
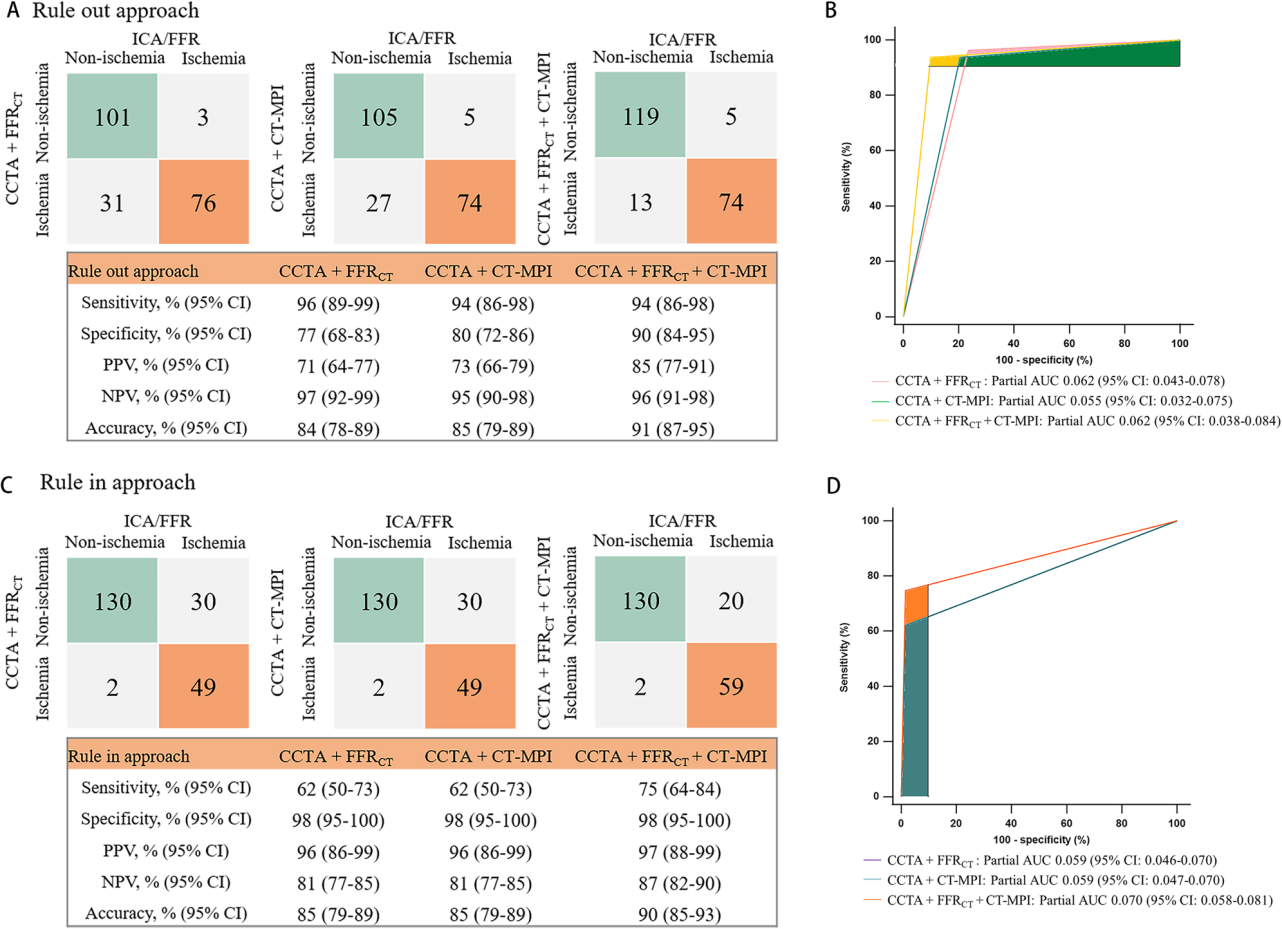
Comparison of diagnostic ability of stepwise strategies for ruling-out and ruling-in functional ischemia. **A**, **B** Confusion matrix and partial AUC of stepwise strategies for ruling-out functional ischemia. **C**, **D** Confusion matrix and partial AUC of stepwise strategies for ruling-in functional ischemia. AUC, area under the receiver-operating characteristic curve; CCTA, coronary computed tomography angiography; CT-MPI, computed tomography myocardial perfusion imaging; FFR_CT_, computed tomography–derived flow fractional reserve; ICA, invasive coronary angiography; FFR, fractional flow reserve; FN, false negative; FP, false positive; TN, true negative; TP, true positive; NPV, negative predictive value; PPV, positive predictive value

The CCTA + FFR_CT_ + CT-MPI stepwise diagnostic algorithm is illustrated in Fig. 3A. The CCTA + FFR_CT_ + CT-MPI stepwise strategy noninvasively diagnosed 88% (185/211) of the stenoses, leaving 12% (26/211) of vessels referring for ICA/FFR. Overall, the diagnostic accuracy was 97% (204/211). For the rule-out approach, the sensitivity and NPV were 94% (95%CI 86–98%) and 96% (95%CI 91–98%), respectively (Fig. 4A). For the rule-in approach, the specificity and PPV were 98% (95%CI 95–100%) and 97% (95%CI 88–99%), respectively (Fig. 4C). This stepwise CCTA + FFR_CT_ + CT-MPI algorithm theoretically avoided invasive FFR measurement in 78% (93/119) of vessels and avoided CT-MPI examinations in 53% (63/119) of vessels.

### Diagnostic performance of the CCTA + FFR_CT _strategy

The stepwise CCTA + FFR_CT_ strategy (Fig. 3B) diagnosed 73% (155/211) of the vessels. Twenty-seven percent (56/211) of the vessels with gray zone FFR_CT_ values would require invasive FFR measurement. Overall, the accuracy was 98%. This stepwise approach theoretically avoided invasive FFR examinations in 53% (63/119) of cases.

### Diagnostic performance of the CCTA + CT-MPI strategy

The stepwise CCTA + CT-MPI strategy (Fig. 3C) could identify 76% (161/211) of vessels noninvasively. Twenty-four percent (50/211) of the vessels with gray zone MBF values are needed for further invasive FFR measurement. The overall accuracy was 97%. This stepwise algorithm avoided invasive FFR examinations in 58% (69/119) of cases.

### Comparison of CCTA + FFR_CT_, CCTA + CT-MPI, and CCTA + FFR_CT_ + CT-MPI stepwise strategy

**Fig. 5:**
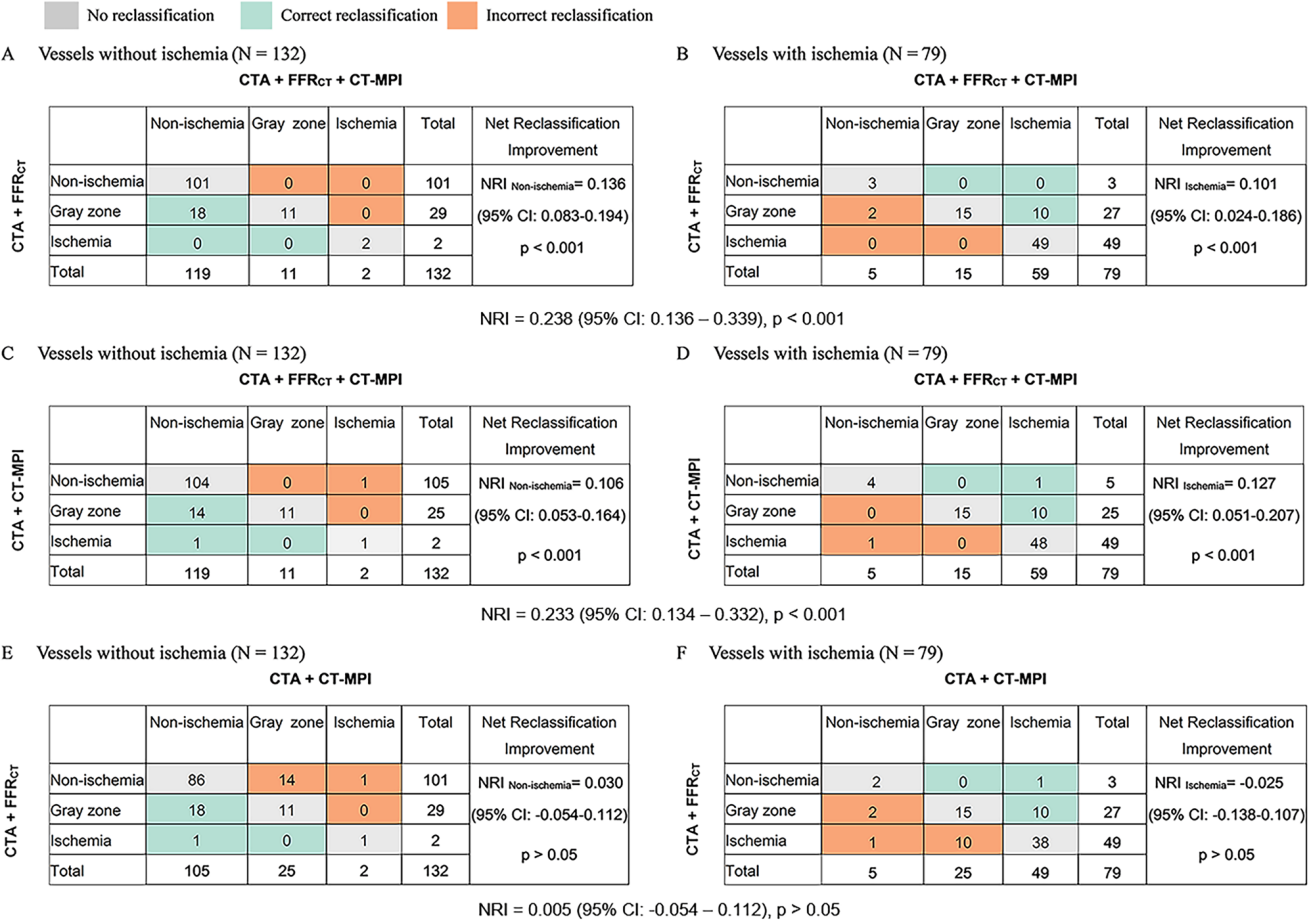
Net reclassification improvement. Reclassification tables: **A, B** CCTA + FFR_CT_ + CT-MPI strategy vs CCTA + FFR_CT_ strategy and (**C, D**) CCTA + FFR_CT_ + CT-MPI strategy vs CCTA + CT-MPI strategy and (**E, F**) CCTA + CT-MPI strategy vs CCTA + FFR_CT_ strategy. The numbers represent the counts of vessels assigned to the indicated risk category. CCTA, coronary computed tomography angiography; CT-MPI, computed tomography myocardial perfusion imaging; FFR_CT_, computed tomography–derived flow fractional reserve; NRI, net reclassification improvement

The CCTA + FFR_CT_ + CT-MPI strategy yielded a higher partial AUC for ruling-in functionally significant lesions than did the CCTA + FFR_CT_ and CCTA + CT-MPI strategies (0.070 [95%CI 0.058–0.081] vs. 0.059 [95%CI 0.046–0.070] vs. 0.059 [95%CI 0.047–0.070], *p* > 0.05) (Fig. 4B). However, the partial AUC did not differ among the ruling-out approach (Fig. 4D). The stepwise CCTA + FFR_CT_ + CT-MPI strategy was superior to CCTA + FFR_CT_, with an NRI of 0.238 (NRI non-ischemia + 0.136, NRI ischemia + 0.101; *p* < 0.001) (Fig. 5A, B), and CCTA + CT-MPI, with an NRI of 0.233 (NRI non-ischemia + 0.106, NRI ischemia + 0.127; *p* < 0.001) (Fig. 5C, D). The NRI between the CCTA + FFR_CT_ and CCTA + CT-MPI strategies was not statistically significant (Fig. 5E, F).

### Sensitivity analyses

Two sensitivity analyses were performed to assess the versatility of the results: (1) The pre-specified algorithm was followed, but the definition of intermediate stenosis on CCTA was set at 40–90% according to the 2021 AHA chest pain guideline. (2) To assess the limitations of vendor-specific FFR_CT_ analysis, sensitivity analysis was performed using a more universal, vendor-independent FFR_CT_ gray zone range (0.75–0.85) based on the literature. Sensitivity analyses produced results basically consistent with main analysis results (Supplementary Table S1, Table S2, Fig. 3, Figure S4).

## Discussion

**Fig. 6:**
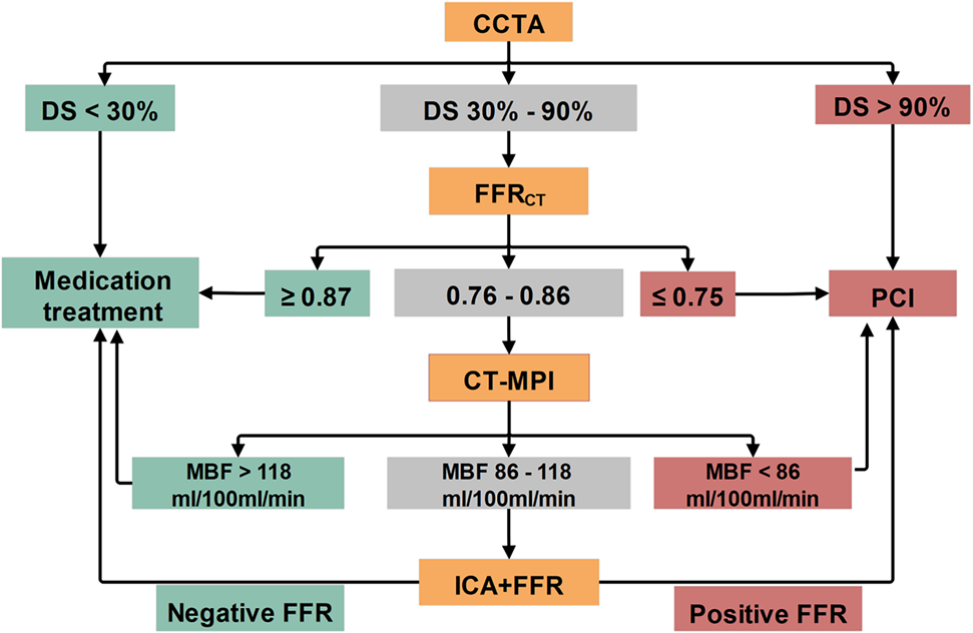
A stepwise strategy based on “one-stop” noninvasive imaging as a gatekeeper of cardiac catheterization. Green color indicates ICA could be delayed safely. Yellow color indicates revascularization is reasonable. Gray color indicates more information is need before revascularization. CCTA, coronary computed tomography angiography; CT-MPI, computed tomography myocardial perfusion imaging; DS, diameter stenosis; FFR_CT_, computed tomography–derived flow fractional reserve; FFR, fractional flow reserve; ICA, invasive coronary angiography; PCI, percutaneous coronary intervention

In summary, a novel CCTA + FFR_CT_ + CT-MPI stepwise strategy (Fig. 6) was validated with clinical patient data. We found that the CCTA + FFR_CT_ + CT-MPI stepwise strategy was superior to the CCTA + FFR_CT_ and CCTA + CT-MPI strategies by minimizing unnecessary invasive diagnostic catheterization without compromising the agreement rate with ICA/FFR. Moreover, the CCTA + FFR_CT_ and CCTA + CT-MPI strategies exhibited comparable performance.

Our findings demonstrated a moderate correlation between FFR_CT_ and invasive FFR (*r* = 0.69, *p* < 001), in accordance with previous studies [12–16]. On average, FFR_CT_ exceeded invasive FFR by 0.03, indicating a low systematic error. Moreover, we reported an FFR_CT_ gray zone of 0.76–0.86, which was generally consistent with that reported in previous studies. Two studies using machine learning–based software reported a FFR_CT_ gray zone range of 0.74–0.85 and 0.74–0.87, respectively [19, 22]. Meta-analyses and reviews have documented more universal, vendor-independent FFR_CT_ gray zone ranges of 0.74–0.82 and 0.75–0.84 [21, 23]. The above evidence may indicate that the concept of the FFR_CT_ gray zone has good generalizability.

We found an MBF gray zone of 86–118 mL/100 mL/min, with an optimal cutoff value of 100 mL/100 mL/min. No literature has reported the gray zone value of MBF, but the cutoff value of MBF observed in our study aligns with previous findings [24, 30]. Of note, many factors contribute to the variability of MBF values, such as heterogeneity of included patients, type of CT scanner, and post-processing software. Accordingly, the range of MBF gray zone reported by our study may only be applicable to scenarios with similar patients and examination protocols.

Each strategy has advantages and disadvantages. Among the three stepwise strategies, the CCTA + FFR_CT_ + CT-MPI stepwise strategy yielded fewer vessels requiring further invasive measurement (12%), whereas the other two strategies yielded similar larger proportions (27%, 24%). The CCTA + CT-MPI strategy was associated with the highest radiation exposure, contrast agent dose, and vasodilator application, whereas the advantage of the CCTA + FFR_CT_ strategy is the no additional imaging acquisitions or administration of vasodilators. The additional time (including image acquisition and post-processing) and expense (including examination and medication cost) for FFR_CT_ and CT-MPI are 5 min and 2000 RMB, and 25 min and 2250 RMB, respectively. As this was a retrospective study, we were unable to perform cost-effectiveness analysis. As is well known, ICA and FFR are associated with high radiation, contrast exposure, and costs. The CCTA + FFR_CT_ + CT-MPI stepwise strategy could potentially improve cost-effectivity by minimizing the need for ICA/FFR.

### Clinical implications

When confronted with CAD, clinicians have difficulty in making correct binary decisions when FFR_CT_ or CT-MPI-derived MBF fell within gray zone threshold. We propose a novel stepwise strategy by which clinicians can better integrate FFR_CT_, CT-MPI, and CCTA. The CCTA + FFR_CT_ + CT-MPI stepwise strategy exhibited high sensitivity, specificity, NPV, and PPV at each step, facilitating greater confidence and accuracy in ICA referral or deferral.

### Limitations

Our study had several limitations. First, our findings are limited by the small cohort sizes and retrospective nature of the analysis in this single-center study. Second, a significant proportion of the participants included in the study exhibited a substantial burden of CAD. Our findings may not be applicable in patients with different prevalence of hemodynamically significant stenosis. Third, since invasive FFR was not performed in all vessels, we could not provide per-patient level analysis and cost-effective analysis. Our findings suggest potential avenues for future research. Further adequately powered prospective randomized studies will be required to validate the proposed stepwise strategy and to determine the cost‐effectiveness of this strategy.

## Conclusions

A CCTA + FFR_CT_ + CT-MPI stepwise strategy is superior to CCTA + FFR_CT_ strategy and CCTA + CT-MPI strategy by minimizing unnecessary invasive diagnostic catheterization while maintaining high rate of agreement with ICA/FFR.

### Supplementary Information

Below is the link to the electronic supplementary material.

Supplementary file1 (PDF 343 KB)

## Abbreviations

AUC: Area under the receiver-operating characteristic curve
CAD: Coronary artery disease
CCTA: Coronary computed tomography angiography
CT-MPI: Computed tomography myocardial perfusion imaging
FFR: Fractional flow reserve
FFR_CT_: Computed tomography–derived flow fractional reserve
ICA: Interventional coronary angiography
MBF: Myocardial blood flow
NPV: Negative predictive value
OMT: Optimal medical treatment
PPV: Positive predictive value

## Declarations

### Guarantor

The scientific guarantor of this publication is Mei Zhang.

### Statistics and biometry

Dr. Chongqi Jia from Shandong University kindly provided statistical advice for this manuscript.

### Study subjects or cohorts overlap

No study subjects or cohorts have been previously reported.

### Methodology

• retrospective

• diagnostic or prognostic study

• performed at one institution
