# Supplementary material for: A stepwise strategy integrating dynamic stress CT myocardial perfusion and deep learning–based FFRCT in the work-up of stable coronary artery disease
Source: Eur Radiol. 2024 Jan 12;34(8):4939–49. doi: 10.1007/s00330-023-10562-x (PMC11254970; doi:10.1007/s00330-023-10562-x)
Supplement: Supplementary file 1 — Supplementary file1 (PDF 343 KB) [file 330_2023_10562_MOESM1_ESM.pdf]

A stepwise strategy integrating dynamic stress CT myocardial perfusion and deep learning based FFRCT in the work-up of stable coronary artery disease

Electronic Supplementary Material

Methods and materials

CCTA and CT-MPI protocol

All participants were scanned with a third-generation DSCT scanner (SOMATOM Force; Siemens Medical Solutions). A comprehensive protocol integrating dynamic CT-MPI and CCTA was employed. Initially, dynamic CT-MPI was started beginning after 3 min of continuous adenosine administration at an infusion rate of 140 μg/kg/min. Iodinated contrast agent (100 mL; Ultravist, 370 mg iodine/mL, Bayer) was given in a bolus injection at a flow rate of 4-5 mL/s, followed by a 40 mL saline flush. Dynamic CT-MPI image acquisitions were started 4 seconds after contrast injection was started. The shuttle-mode acquisition technique was used to image the complete left ventricle. By moving the table back and forth after each acquisition, 2 series of images were collected, which together covered the entire myocardium. Dynamic image acquisition was performed in systole 250 ms after the R-wave. Depending on heart rate, scans were launched every second or third heart cycle, resulting in a series of 10–15 data samples acquired over a period of approximately 32 seconds. The following scan parameters were used: collimation = 192 x 0.6 mm, gantry rotation time = 250 ms, temporal resolution = 66 ms, shuttle-mode z-axis coverage of 105 mm, tube voltage = 70 kV, and

automated tube current scaling. CARE kV and CARE dose 4D were used as reference.

Nitroglycerin was given sublingually to all participants 5 min after CT-MPI. CCTA was performed by using a bolus tracking technique, with regions of interest (ROIs) placed in the ascending aorta. Subsequently, a bolus of contrast media was injected into the antecubital vein at a rate of 4-5 ml/s, followed by a 40-mL saline flush using a dual-barrel power injector. Retrospective electrocardiography-triggered sequential acquisition was carried out in all patients undergoing CCTA, with the center of the triggering window set at diastole or systole, depending on the heart rate. The reference tube voltage and current were set as 100 kV and 320 mAs (CARE kV and CARE dose 4D were used as reference). The entire protocol took approximately 15-20 minutes.

CCTA assessments and Dynamic CT-MPI assessments

Images were reconstructed with a smooth kernel (Bv36) and third generation iterative reconstruction (IR) technique (strength 3, ADMIRE, Siemens), slice thickness of 0.75 mm, and an increment of 0.5 mm. For quantitative analysis, CCTA images were analyzed on an offline workstation (Syngo Via, Siemens) by two experienced radiologists who were blinded to all other modalities. The degree of stenosis was reported as the percentage of lumen diameter reduction. In case of disagreement, a third radiologist possessing greater expertise was consulted, and a consensus was reached by discussion. Segmental analysis of the coronary arteries was performed for all arteries > 1.5 mm in diameter. A coronary lesion with ≥ 50% stenosis with a diameter greater than 1.5 mm was defined as having obstructive CAD. In addition, patients with poor CT image quality were excluded.

The CT-MPI images were processed with the CT-MPI software package (VPCT, Siemens) and

reconstructed using a dedicated kernel to reduce iodine beam-hardening artifacts (b23f, Qr36). Motion correction was performed for all images. The influx of contrast medium was measured using the arterial input function (AIF). The AIF was sampled in the descending aorta by including both cranial and caudal sections. MBF was derived from the time attenuation curves for each voxel. A dedicated semiautomated parametric deconvolution algorithm based on a 2-compartment model of intra- and extravascular space was used. MBF was calculated as the ratio between the maximum slope of the fit curve and the peak AIF. To sample the MBF, the region of interest (ROI) was manually placed on a short-axis view on a segment basis according to the American Heart Association (AHA) 17-segment model. The ROI was placed to cover the whole segment without perfusion defects or cover the area (at least 50 mm^2) of suspected perfusion defects within the segment. According to the AHA recommendation, individual myocardial segments were assigned to the 3 major coronary artery territories and were adjusted for differences in dominance. Two experienced radiologists who were blinded to the participants' clinical history independently analyzed the CT-MPI data. For quantitative analysis, in stenosis-subtended territories, the absolute MBF was calculated as the mean value of MBF for segments with perfusion defects. Within reference territories, the absolute MBF was calculated as the mean value of MBF for all segments assigned to this territory.

CCTA and CT-MPI image quality was assessed with a 4-point scale (1 = poor; 2 = moderate; 3 = good; 4 = excellent). CCTA and CT-MPI images with poor image quality were excluded from the analysis.

Machine learning based FFR_{CT} assessments

Deep learning-based FFR was computed by using a novel tree-structured recurrent neural network solution (TreeVes-Net). The framework was trained using a database of 13,000 synthetic coronary trees based on the geometric parameters of the coronary trees and was validated using a database of 180 real coronary trees with invasive FFR measurements. In the training database, the values of the geometric parameters were randomly prespecified in the appropriate ranges. The input to TreeVes-Net is the hydrodynamics-related geometric feature vector including local vascular features, local stenotic features, and global features for one position at the centerline of the coronary artery extracted from the input image of these synthetic coronary arteries. The distribution of FFR values along the coronary centerline is calculated by solving the Navier–Stokes equations with the finite element method. In TreeVes-Net, a fully connected multilayer neural network was used to embed the input image features, and bidirectional recursive neural network with long short-term memory was used to tackle the problem of spatial long-term dependency among fluid states at different points on coronary centerlines. For the output of the results, 3D color-coded coronary maps were generated to visualize the outcome. The lesion-specific FFR_{CT} values were measured 20 mm distal to the stenosis.

Table S1 Sensitivity analysis of the diagnostic ability of stepwise strategies (intermediate diameter stenosis was defined as 40%-90%, and the grey zone of FFR_{CT} value was defined as 0.76-0.86).

	Stepwise strategy1 (CCTA + FFR_{CT})	Stepwise strategy 2 (CCTA + MBF)	Stepwise strategy 3 (CCTA + FFR_{CT} + MBF)
Vessels with functional ischemia, 79 (37%)			
Rule out approach			
TP/TN/FP/FN	73/109/23/6	72/110/22/7	72/120/12/7
Sensitivity, % (95% CI)	91 (83-96)	91 (83-96)	91 (83-96)
Specificity, % (95% CI)	83 (75-89)	83 (76-89)	91 (85-95)
PPV, % (95% CI)	76 (69-82)	77 (69-83)	86 (78-91)
NPV, % (95% CI)	94 (88-97)	94 (89-97)	94 (89-97)
Accuracy, % (95% CI)	86 (80-90)	86 (81-91)	91 (86-94)
Rule in approach			
TP/TN/FP/FN	49/130/2/30	49/131/1/30	59/130/2/20
Sensitivity, % (95% CI)	62 (50-73)	62 (50-73)	75 (64-84)
Specificity, % (95% CI)	98 (95-100)	99 (96-100)	98 (95-100)
PPV, % (95% CI)	96 (86-99)	98 (87-100)	97 (88-99)
NPV, % (95% CI)	81 (77-85)	81 (77-85)	87 (82-90)
Accuracy, % (95% CI)	85 (79-89)	85 (80-90)	90 (85-93)
Gray zone, n (%)	44 (21)	43 (20)	22 (10)

Data are n (%) or percentage, unless otherwise specified. CCTA, coronary computed tomography angiography; CT-MPI, computed tomography myocardial perfusion imaging; FFRCT, computed tomography derived flow fractional reserve; ICA, invasive coronary angiography; FFR, fractional flow reserve; FN, false negative; FP, false positive; TN, true negative; TP, true positive; NPV, negative predictive value; PPV, positive predictive value.

Table S2 Sensitivity analysis of the diagnostic ability of stepwise strategies (intermediate diameter stenosis was defined as 30%-90%, and the grey zone of FFR_{CT} value was defined as 0.75-0.85).

	Stepwise strategy1 (CCTA + FFR_{CT})	Stepwise strategy 2 (CCTA + MBF)	Stepwise strategy 3 (CCTA + FFR_{CT} + MBF)
Vessels with functional ischemia, 79 (37%)			
Rule out approach			
TP/TN/FP/FN	73/111/20/6/	74/105/27/5	71/120/12/8
Sensitivity, % (95% CI)	92 (84-97)	94 (86-98)	90 (81-96)
Specificity, % (95% CI)	84 (77-90)	80 (72-86)	91 (85-95)
PPV, % (95% CI)	78 (70-84)	73 (66-79)	86 (77-91)
NPV, % (95% CI)	95 (90-98)	95 (90-98)	94 (89-97)
Accuracy, % (95% CI)	87 (82-91)	85 (79-89)	91 (86-94)
Rule in approach			
TP/TN/FP/FN	43/130/2/36	49/130/2/30	53/130/2/26
Sensitivity, % (95% CI)	54 (43-66)	62 (50-73)	67 (56-77)
Specificity, % (95% CI)	98 (95-100)	98 (95-100)	98 (95-100)
PPV, % (95% CI)	96 (84-99)	96 (86-99)	96 (87-99)
NPV, % (95% CI)	78 (74-82)	81 (77-85)	83 (78-87)
Accuracy, % (95% CI)	82 (76-87)	85 (79-89)	87 (81-91)
Gray zone, n (%)	51 (24)	50 (24)	27 (13)

Data are n (%) or percentage, unless otherwise specified. CCTA, coronary computed tomography angiography; CT-MPI, computed tomography myocardial perfusion imaging; FFRCT, computed tomography derived flow fractional reserve; ICA, invasive coronary angiography; FFR, fractional flow reserve; FN, false negative; FP, false positive; TN, true negative; TP, true positive; NPV, negative predictive value; PPV, positive predictive value.

Figure S1 Relationship between CCTA, FFRCT and CT-MPI and presence of functionally ischemia identified by ICA/FFR.

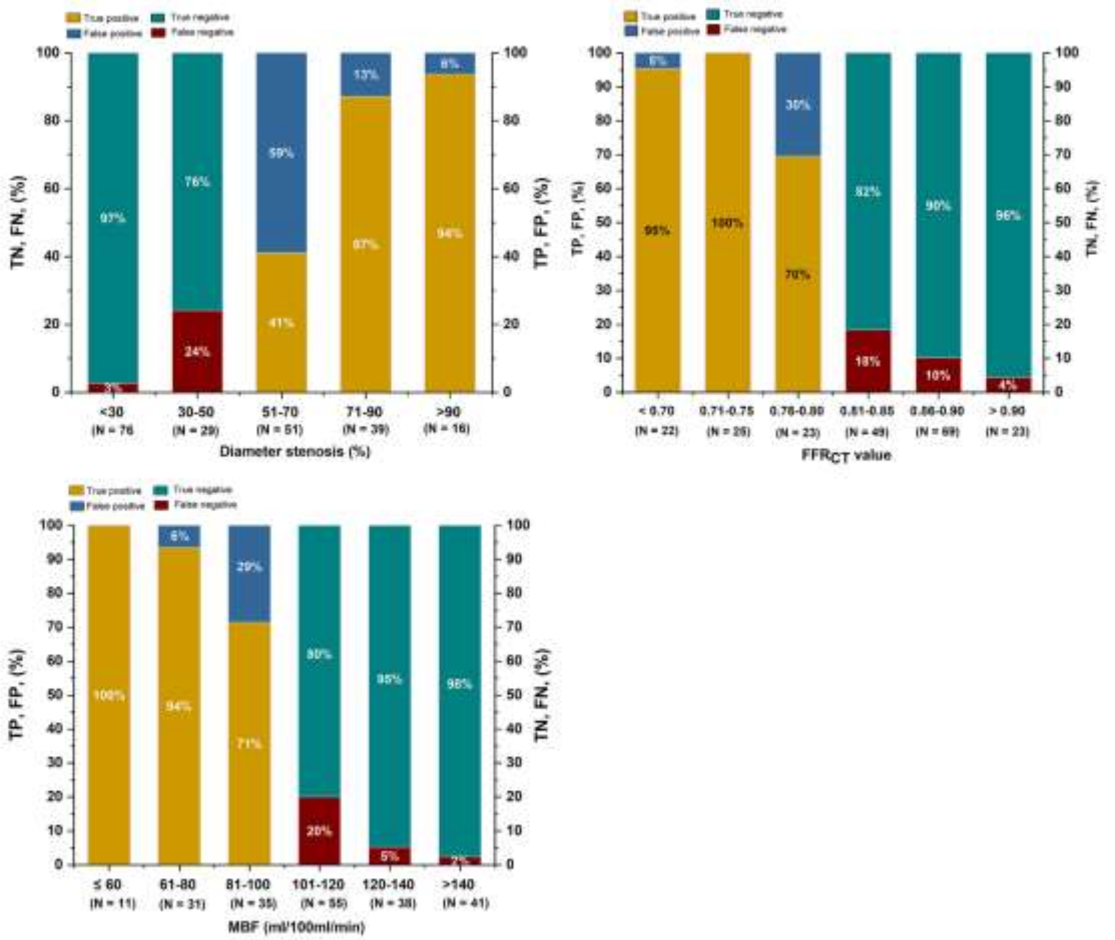

CCTA, FFRCT, and CT-MPI can rule in or rule out ischemia with almost completely certainty at extremely low or high values. For each imaging modality, the highest frequency of false positive and false negative cases was clustered around the cutoff value. FFRCT, computed tomography derived flow fractional reserve; FFR, fractional flow reserve; FP, false positive; FN, false negative; ICA, invasive coronary angiography; MBF, myocardial blood flow; TP, true positive; TN, true negative.

Figure S2 Correlation analysis and Bland–Altman Plots.

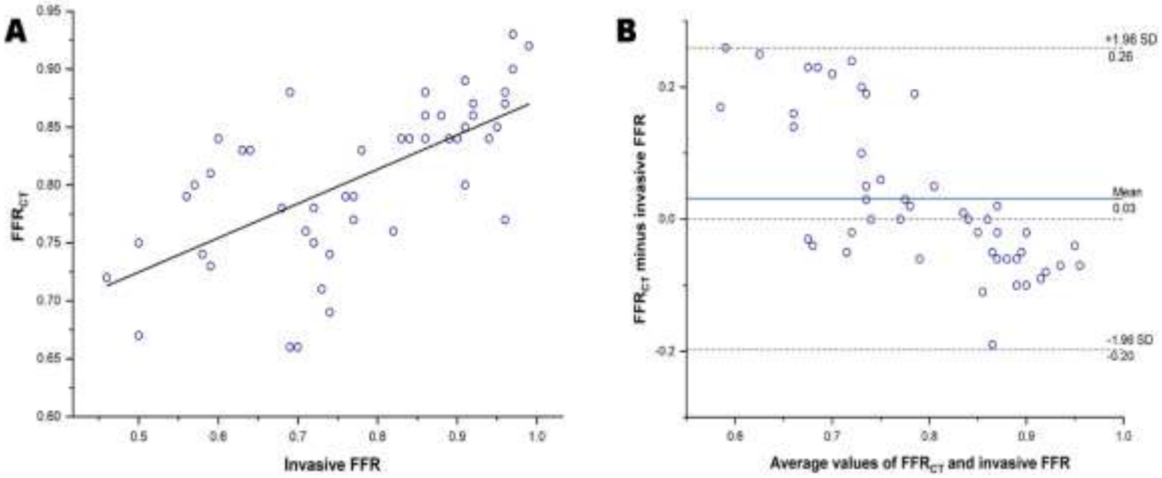

(A) Spearman correlation analysis shows a moderate correlation between FFR_{CT} and invasive FFR with a coefficient of correlation of 0.69 ($p < 0.001$). (B) The mean difference between FFR_{CT} and invasive FFR was 0.03 (95% CI: -0.003-0.067), showing slight systematic overestimation of FFR by FFR_{CT}. SD, standard deviation; other abbreviations are shown in Figure S1.

Figure S3 Flowchart algorithm for the stepwise strategy: Sensitive analysis I (intermediate diameter stenosis was defined as 40%-90%, and the grey zone of FFRCT value was defined as 0.76 - 0.86).

No reclassification | Correct reclassification | Incorrect classification

A. Vessels without ischemia (N = 132)

Columns: CTA + FFR_{CT} + CT-MPI; rows: CTA + FFR_{CT}

	Non-ischemia	Gray zone	Ischemia	Total	Net Reclassification Improvement
Non-ischemia	109	0	0	109	NRI $_{Non\text{-}ischemia}$= 0.083
Gray zone	11	10	0	21	(95% CI: 0.049-0.164)
Ischemia	0	0	2	2	p < 0.001
Total	120	10	2	132	

B. Vessels with ischemia (N = 79)

Columns: CTA + FFR_{CT} + CT-MPI; rows: CTA + FFR_{CT}

	Non-ischemia	Gray zone	Ischemia	Total	Net Reclassification Improvement
Non-ischemia	6	0	0	6	NRI $_{Ischemia}$= 0.114
Gray zone	1	13	10	24	(95% CI: 0.071-0.236)
Ischemia	0	0	49	49	p < 0.001
Total	7	13	59	79	

NRI = 0.197 (95% CI: 0.107 – 0.306), p < 0.001

C. Vessels without ischemia (N = 132)

Columns: CTA + FFR_{CT} + CT-MPI; rows: CTA + CT-MPI

	Non-ischemia	Gray zone	Ischemia	Total	Net Reclassification Improvement
Non-ischemia	109	0	1	110	NRI $_{Non\text{-}ischemia}$= 0.076
Gray zone	11	10	0	21	(95% CI: 0.035-0.114)
Ischemia	0	0	1	1	p < 0.05
Total	120	10	2	132	

D. Vessels with ischemia (N = 79)

Columns: CTA + FFR_{CT} + CT-MPI; rows: CTA + CT-MPI

	Non-ischemia	Gray zone	Ischemia	Total	Net Reclassification Improvement
Non-ischemia	6	0	1	7	NRI $_{Ischemia}$= 0.127
Gray zone	0	13	10	23	(95% CI: 0.062-0.207)
Ischemia	1	0	48	49	p < 0.001
Total	7	13	59	79	

NRI = 0.203 (95% CI: 0.124 – 0.323), p < 0.001

E. Vessels without ischemia (N = 132)

Columns: CTA + CT-MPI; rows: CTA + FFR_{CT}

	Non-ischemia	Gray zone	Ischemia	Total	Net Reclassification Improvement
Non-ischemia	98	11	0	109	NRI $_{Non\text{-}ischemia}$= 0.008
Gray zone	11	10	0	21	(95% CI: -0.075-0.124)
Ischemia	1	0	1	2	p > 0.05
Total	110	21	1	132	

F. Vessels with ischemia (N = 79)

Columns: CTA + CT-MPI; rows: CTA + FFR_{CT}

	Non-ischemia	Gray zone	Ischemia	Total	Net Reclassification Improvement
Non-ischemia	5	0	1	6	NRI $_{Ischemia}$= -0.013
Gray zone	1	13	10	24	(95% CI: -0.159-0.137)
Ischemia	1	10	38	49	p > 0.05
Total	7	23	49	79	

NRI = -0.005 (95% CI: -0.094 – 0.102), p > 0.05

CCTA, coronary computed tomography angiography; CT-MPI, computed tomography myocardial perfusion imaging; DS, diameter stenosis; FFR_{CT}, computed tomography derived flow fractional reserve; FFR, fractional flow reserve; ICA, invasive coronary angiography.

Figure S4 Flowchart algorithm for the stepwise strategy: Sensitive analysis II (intermediate diameter stenosis was defined as 30%-90%, and the grey zone of FFRCT value was defined as 0.75 - 0.85).

No reclassification Correct reclassification Incorrect classification

A. Vessels without ischemia (N = 132)

CTA + FFR_{CT} + CT-MPI

CTA + FFR_{CT}	Non-ischemia	Gray zone	Ischemia	Total	Net Reclassification Improvement
Non-ischemia	111	0	0	111	$NRI_{Non-ischemia}$= 0.068
Gray zone	9	10	0	19	(95% CI: 0.013-0.106)
Ischemia	0	0	2	2	p < 0.05
Total	120	10	2	132	

B. Vessels with ischemia (N = 79)

CTA + FFR_{CT} + CT-MPI

CTA + FFR_{CT}	Non-ischemia	Gray zone	Ischemia	Total	Net Reclassification Improvement
Non-ischemia	6	0	0	6	$NRI_{Ischemia}$= 0.114
Gray zone	2	17	11	30	(95% CI: 0.064-0.197)
Ischemia	0	0	43	43	p < 0.001
Total	8	17	54	79	

NRI = 0.182 (95% CI: 0.103 – 0.282), p < 0.001

C. Vessels without ischemia (N = 132)

CTA + FFR_{CT} + CT-MPI

CTA + CT-MPI	Non-ischemia	Gray zone	Ischemia	Total	Net Reclassification Improvement
Non-ischemia	104	0	1	105	$NRI_{Non-ischemia}$= 0.106
Gray zone	15	10	0	25	(95% CI: 0.053-0.164)
Ischemia	1	0	1	2	p < 0.001
Total	120	10	2	132	

D. Vessels with ischemia (N = 79)

CTA + FFR_{CT} + CT-MPI

CTA + CT-MPI	Non-ischemia	Gray zone	Ischemia	Total	Net Reclassification Improvement
Non-ischemia	5	0	0	5	$NRI_{Ischemia}$= 0.063
Gray zone	1	17	8	26	(95% CI: 0.011-0.107)
Ischemia	2	0	46	48	p < 0.05
Total	8	17	54	79	

NRI = 0.169 (95% CI: 0.097 – 0.273), p < 0.001

E. Vessels without ischemia (N = 132)

CTA + CT-MPI

CTA + FFR_{CT}	Non-ischemia	Gray zone	Ischemia	Total	Net Reclassification Improvement
Non-ischemia	95	15	1	111	$NRI_{Non-ischemia}$= -0.045
Gray zone	9	10	0	19	(96% CI: -0.096-0.101)
Ischemia	1	0	1	2	p > 0.05
Total	105	25	2	132	

F. Vessels with ischemia (N = 79)

CTA + CT-MPI

CTA + FFR_{CT}	Non-ischemia	Gray zone	Ischemia	Total	Net Reclassification Improvement
Non-ischemia	3	1	2	6	$NRI_{Ischemia}$= 0.051
Gray zone	2	17	11	30	(95% CI: -0.073-0.106)
Ischemia	0	8	35	43	p > 0.05
Total	5	26	48	79	

NRI = 0.006 (95% CI: -0.071 – 0.102), p > 0.05

CCTA, coronary computed tomography angiography; CT-MPI, computed tomography myocardial perfusion imaging; DS, diameter stenosis; FFRCT, computed tomography derived flow fractional reserve; FFR, fractional flow reserve; ICA, invasive coronary angiography.
